# Perioperative management of a patient undergoing resection of interleukin-6 producing pheochromocytoma

**DOI:** 10.1186/s40981-021-00449-5

**Published:** 2021-06-11

**Authors:** Masahiro Kuroki, Hiroto Suzuki, Misato Kurota, Masaki Nakane, Kaneyuki Kawamae

**Affiliations:** 1 Department of Anesthesiology, Yamagata University Hospital, 2-2-2 Iida-nishi, Yamagata City, Yamagata, 990-9585 Japan; 2 Critical Care Center, Yamagata University Hospital, 2-2-2 Iida-nishi, Yamagata City, Yamagata, 990-9585 Japan; 3 Department of Emergency and Critical Care Medicine, Yamagata University Hospital, 2-2-2 Iida-nishi, Yamagata City, Yamagata, 990-9585 Japan

**Keywords:** Pheochromocytoma, Interleukin-6, Inflammatory

## Abstract

**Background:**

Pheochromocytomas produce hormones, cytokines, and catecholamines. We report perioperative anesthetic management of a rare interleukin-6 (IL-6)-producing pheochromocytoma.

**Case presentation:**

A 32-year-old female was scheduled for laparoscopic adrenalectomy for pheochromocytoma. She had a sustained high fever with elevated serum noradrenaline and IL-6 levels. The persistent high inflammatory state, followed by anemia, malnutrition, and coagulopathy, led us to prepone her date of surgery. During general anesthesia, alpha-blockers allowed hemodynamic fluctuations to be controlled. During surgery, simultaneous increases in noradrenaline and IL-6 levels were observed, which rapidly declined after tumor removal. Her postoperative course was uneventful, without pulmonary edema caused by cytokine storms.

**Conclusions:**

The conventional pheochromocytoma strategy allowed surgical removal of an IL-6-producing pheochromocytoma under general anesthesia. However, management of high inflammatory states that induce anemia and coagulopathy is necessary.

## Background

Pheochromocytomas are rare tumors with an incidence of 2–8 per 1,000,000 [1]. Pheochromocytomas have the unique ability to produce various hormones and cytokines as well as catecholamines [2]. Interleukin-6 (IL-6)-producing pheochromocytomas reportedly cause high inflammatory states and induce coagulopathy and anemia [3]. In addition, IL-6 levels may be suppressed by non-steroidal anti-inflammatory drugs (NSAIDs) and alpha- and beta-blockers [4–6]. However, no literature describes perioperative management, including anesthesia technique and its association with IL-6-related changes during surgery. This report presents the perioperative anesthetic management and the results of catecholamine and cytokine measurements of a patient with a pheochromocytoma.

## Case presentation

A 32-year-old, 70 kg, 155 cm female presented with a one-month history of fever, which she treated like a common cold. Her fever did not improve. Further examination showed elevated blood and urinary noradrenaline levels. We additionally noted increased uptake of iodine-123-metaiodobenzylguanidine in the left adrenal glands, leading to the diagnosis of pheochromocytoma.

On admission, she presented with a headache and general malaise. Her blood pressure (BP) was 126/76 mmHg, heart rate (HR) was 113 beats/min, and body temperature (BT) was 38.5 °C. Chest radiography results were unremarkable. Transthoracic echocardiography revealed no left ventricular hypertrophy. Magnetic resonance imaging revealed a 70-mm left adrenal gland mass. Laboratory tests revealed a high inflammatory state with the following results: a white blood cell count of 12,400/μl; C-reactive protein (CRP) levels at 27.9 mg/dL; anemia, coagulopathy, and malnutrition, with hemoglobin at 8.9 g/dl; a prothrombin time-international normalized ratio (PT-INR) of 1.49; and albumin levels at 2.1 g/dL. For catecholamines, there was a significant increase in serum noradrenaline (2.5 ng/ml [normal range 0.15–0.57]). Serum adrenaline (< 0.01 ng/mL) and dopamine levels (0.04 ng/mL) were almost normal. In addition, serum IL-6 (78.8 pg/mL [normal range < 4]) was elevated, while IL-1β (10 pg/mL) and tumor necrosis factor-alpha (TNF-α; 1.17 pg/mL) levels were normal, which led us to diagnose an IL-6-producing pheochromocytoma. Laparoscopic left adrenal tumor resection was subsequently scheduled (Table 1).

**Table 1:** Laboratory data pre-and post-operation

	Pre-operation	Post-operation day1	Post-operation day7	Normal values
WBC (/μl)	12400	8660	5320	3300–8600
CRP (mg/dl)	27.88	11.05	0.4	< 0.14
IL-6 (pg/ml)	78.8	17.1	1.9	< 4
Adrenaline (ng/ml)	< 0.01	< 0.01	< 0.01	< 0.017
Noradrenaline (ng/ml)	2.5	0.85	0.18	0.15-0.57
Dopamine (ng/ml)	0.04	0.03	< 0.02	< 0.03

*WBC* white blood cell, *CRP* C-reactive protein, *IL-6* interleukin-6

**Fig. 1:**
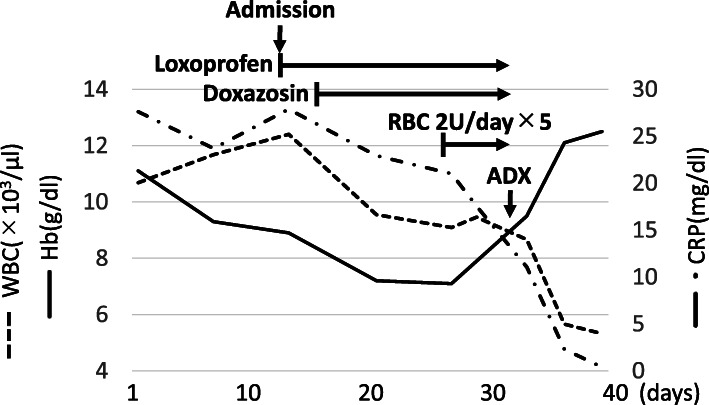
WBC, Hb, and CRP levels before and after therapy. WBC, white blood cell; Hb, hemoglobin; CRP, C-reactive protein; RBC, red blood cell; ADX, adrenalectomy

After admission, oral loxoprofen administration was started to control systemic inflammation. We also initiated oral doxazosin, a common preoperative treatment for pheochromocytoma. The surgery was scheduled to take place after approximately 2 months to allow for incremental increases of the doxazosin dose. However, her persistently high inflammatory markers, anemia, malnutrition, and coagulopathy, led us to prepone her surgery. The dosage of doxazosin peaked at 12 mg. We did not reach 16 mg, as initially planned, because there were only 17 days before surgery. Anemia due to continued systemic inflammation was also prominent, and two units of packed red blood cells were administered daily starting 5 days before surgery (Fig. 1).

**Fig. 2:**
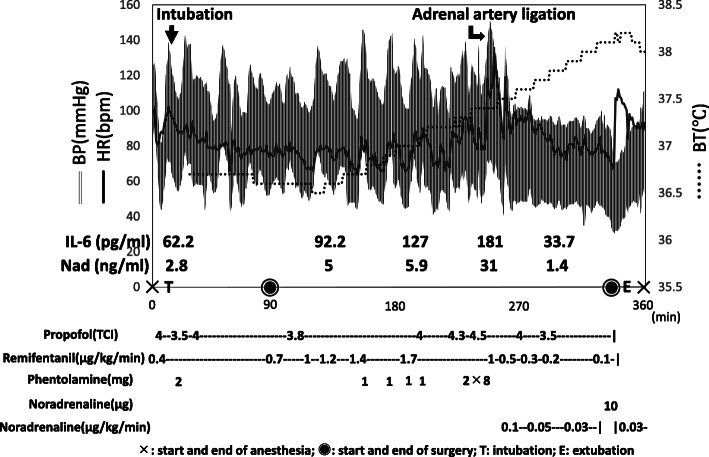
Anesthesia record and serum IL-6, noradrenaline levels during surgery. BP, blood pressure; HR, heart rate; BT, body temperature. IL-6, interleukin-6; Nad, noradrenaline

Her BP was 111/68 mmHg on the day of surgery, HR was 95 beats/min, without arrhythmias, and BT was 37.0 °C. General anesthesia was performed, and an erector spinae plane block was used for perioperative analgesia. Epidural anesthesia was not performed because of coagulopathy. In addition to the standard monitors, an arterial catheter was placed in the radial artery. General anesthesia was induced with target-controlled infusion (TCI) of an effect-site propofol concentration set at 4.0 μg/ml, remifentanil 0.4 μg/kg/min, and rocuronium 50 mg. There were no hemodynamic fluctuations after tracheal intubation. Her BP surged to 146/79 mmHg when she was placed in a head-down position to insert the central venous catheter into the right internal jugular vein. Consequently, phentolamine (2 mg) was immediately administered. Flurbiprofen (50 mg) was administered before the start of surgery. After the start of pneumoperitoneum, the infusion rate of remifentanil was increased to 1.0–1.7 μg/kg/min, with 1 mg of phentolamine administered as appropriate. Meanwhile, her systolic BP was kept around 140 mmHg. During direct tumor manipulation, an increase in BP of 150/70 mmHg and tachycardia of 115 beats/min were observed, which required a bolus infusion of phentolamine (2 mg) for a total of 12 mg. Beta-blocker administration and continuous infusion of phentolamine were not necessary to control hemodynamic fluctuations. After tumor removal, the surgery was completed with a continuous infusion of noradrenaline 0.05–0.1 μg/kg/min. During the operation, the patient’s BT rose to 38.2 °C without a warmer and quickly dropped to 37 °C postoperatively. The duration of surgery and anesthesia time were 232 min and 361 min, respectively, with a volume of infusion of 2,200 mL, a blood loss of 50 mL, and a urine output of 1,450 mL (Fig. 2).

Postoperatively, she was transferred to the intensive care unit, and a continuous infusion of 0.05 μg/kg/min noradrenaline was administered. This was eventually discontinued, and the patient moved to the general ward the next day. On postoperative day (POD) 1, chest radiography was normal without any findings of vascular hyperpermeability. Laboratory tests showed improved inflammatory markers. Further, her noradrenaline and IL-6 levels decreased, which normalized by POD7 (Table 1). The patient was discharged with an uneventful postoperative course on POD8.

## Discussion

We performed perioperative and anesthetic management for a patient with an IL-6-producing pheochromocytoma. In general, preoperative management of patients with pheochromocytoma includes alpha-blockers, and it has been shown to reduce perioperative mortality from 13–45% to 0–3% [7]. Our patient’s dose of doxazosin could not be increased to the planned 16 mg/day when her surgery date was moved up. Alpha-blocker therapy was continued for 17 days right up to the day before surgery. Generally, 7–14 days are recommended to prevent a hypertensive crisis caused by a catecholamine surge from the tumor [1]. Furthermore, intraoperative complications are reportedly rare after 10 days of the preoperative administration of alpha-blockers [8]. In addition, given that all the Roizen criteria for judging alpha-blocker efficacy were met in this case [9], the timing of the surgery was believed appropriate. In fact, only bolus infusions of phentolamine were necessary to allow the operation to be completed without a hypertensive crisis.

For patients with IL-6-producing pheochromocytoma, the preoperative administration of NSAIDs and alpha- and beta-blockers reportedly improves inflammation by decreasing or normalizing serum IL-6 levels [4, 5]. Although our patient’s inflammatory markers were reduced somewhat, a high inflammatory state with anemia, coagulopathy, and malnutrition remained and continued to progress. These symptoms and the patient's complaints of general malaise resulted in an earlier surgery. These are not found in pheochromocytomas that produce only catecholamines and appear to be specific to IL-6-producing pheochromocytomas. IL-6 induces hepcidin and causes secondary anemia, promoting CRP production and inhibiting albumin production [10, 11]. It also increases the expression of monocyte tissue factors, resulting in coagulopathy [12, 13]. Therefore, although it has been reported that epidural anesthesia reduces postoperative complications in adrenalectomy, it could not be performed in this case [14].

We used propofol as an anesthetic agent. It has been reported that intraoperative serum IL-6 levels were lower in general anesthesia compared with desflurane [15]. Moreover, it suppressed the release of inflammatory cytokines by lipopolysaccharides [16]; hence, propofol was selected in this case. However, the effect on IL-6 released directly from tumors is unknown, and its role as an anti-inflammatory agent is unclear. In addition, D2 dopamine receptor antagonists used as antiemetics, such as metoclopramide and droperidol, release catecholamines from pheochromocytoma [17]. Although it is unclear whether IL-6 is released simultaneously with catecholamines, in this case, these drugs should be avoided. Propofol is associated with a lower frequency of postoperative nausea and vomiting (PONV) than inhalational anesthetics [18]. For that reason, it was chosen as the anesthetic agent. In fact, there was no PONV in our patient. Finally, flurbiprofen was administered before the start of surgery, hoping that it would have an anti-inflammatory effect. However, it failed to suppress the increase in body temperature in this case.

In summary, we conducted the perioperative anesthetic management of a patient with an IL-6-producing pheochromocytoma. Anesthesia can be completed using the same management course as conventional pheochromocytoma; however, individual management, including early surgery, anemia, and coagulopathy, is necessary. In addition, intraoperative measurement of catecholamines and cytokines indicated that IL-6 was produced by the tumor.

## Data Availability

Data relevant to this case report are not available for public access because of patient privacy concerns.

## Abbreviations

IL-6: Interleukin-6
NSAIDs: Non-steroidal anti-inflammatory drugs
BP: Blood pressure
HR: Heart rate
BT: Body temperature
CRP: C-reactive protein
PT-INR: Prothrombin time-international normalized ratio
TNF-α: Tumor necrosis factor-alpha
TCI: Target-controlled infusion
POD: Postoperative day
PONV: Postoperative nausea and vomiting
